# Consumptive Cases in Convalescent and Other Homes

**Published:** 1901-11-30

**Authors:** 


### CONSUMPTIVE CASES IN CONVALESCENT AND OTHER HOMES.

A PROTEST BY HELEN TODD, MATRON, NATIONAL SANATORIUM, BOURNEMOUTH.

There are various societies and associations in this country whose chief object is the welfare of poor working girls. The two largest of these organisations are "The Girls' Friendly Society"—by means of which the Church of England seeks to protect and influence for good her girl members, especially those living away from home in service or at business—and the undenominational "Young Women's Christian Association" which strives to band the girls together for mutual help towards living a cleanly, moral, and Christian life. One great point of difference between the two societies lies in their scheme of government; that of the G.F.S. consists of a central authority at headquarters and local committees which manage the branches in their own districts; the branches are further grouped together for administrative purposes under diocesan councils or committees which form a link between the central authority and the scattered branches. The local committees upon whom devolves the management of these branches are mainly composed of the clergy, their wives, and ladies of education and culture interested in women's work.

The different branches of the Y.W.C.A. are much more independent than those of the G.F.S., and their local committees are composed to a great extent of the girls themselves, in whose interests the branches are founded.

The composition of these committees is important, as the minutiæ of details in the carrying on of homes of rest, etc., are very largely in their hands, and it is with the hope of calling their attention to a much-needed reform in management of branches that the following criticisms are made and suggestions submitted to meet the difficulty.

Both the G.F.S. and Y.W.C.A. have one great feature in common, *i.e.* the provision and maintenance of homes where girls in business or out of a situation may lodge and board at a very low cost under the personal supervision of an experienced "Matron" who will mother them generally and look after their health and morals. These homes, which are variously known as "Lodges," "Institutes," and "Homes of Rest," are all arranged on much the same plan. A common sitting-room, a general dining-room, and bedrooms shared by several members, the number in each bedroom varying according to its size.

The majority of these homes have been originally built for private houses and since adapted to their present use, and rents being high and the girls' payments cut down to the lowest figure that will pay expenses, private rooms for members are out of the question.

There is therefore a very grave element of danger in admitting girls suffering from consumption into such homes. Cases have not hitherto been uncommon when girls waiting for admission into sanatoria have been received into the boarding houses, girls sometimes suffering from phthisis in an advanced stage of the disease and who have no knowledge of the precautions necessary to ensure the safety of those with whom they live.

It must be remembered that most of the boarders in these institutions are either servants whose health compels them to take a rest between two situations, or shop assistants, whose daily work lies in a hot and vitiated atmosphere, and whose lungs are therefore frequently in a delicate condition, supplying a favourable soil for the growth of the tubercle bacillus. Moreover, the conditions of shop life do not tend to induce a great love of open windows at night, and in spite of the more general teaching of the laws of health, the great majority of both servant girls and shop assistants still sleep in stuffy rooms with tightly-closed windows.

Health and power to work are too often the only stock in trade of these girls, and inquiries will often reveal that they have relations to support with their scanty earnings.

How grave then is the responsibility of those who permit these ignorant young women to run the undoubted risk of contracting a long, tedious, and expensive disease by introducing a possible source of infection amongst them! It is surely the duty of all members of committees managing such homes to examine closely their rules and regulations, to take all precautions against the danger, and to recognise it as one that cannot be ignored with impunity. It might be well if all girls were required to send a medical certificate of their health to the superintendent of the home when applying for admission, and surely the superintendents could be authorised to obtain a skilled medical opinion in any case which might appear suspicious during residence in the institution. On no account should a consumptive girl be allowed to share a bedroom with another member, and every possible means should be taken of teaching both the sick and healthy the importance of the proper disposal of sputa

(even in cases of supposed "ordinary cold"), the absolute necessity of the proper ventilation of sleeping rooms, and other simple rules of hygiene.

